# Current and potential providers of blood pressure self-screening: a mixed methods study in Oxfordshire

**DOI:** 10.1136/bmjopen-2016-013938

**Published:** 2017-03-22

**Authors:** A C Tompson, S G Fleming, C J Heneghan, R J McManus, S M Greenfield, F D R Hobbs, A M Ward

**Affiliations:** 1 Nuffield Department of Primary Care Health Sciences, University of Oxford, Oxford, UK; 2 Institute of Applied Health Research, University of Birmingham, Birmingham, UK

**Keywords:** PRIMARY CARE, screening, self-monitoring, mixed-methods

## Abstract

**Objectives:**

To (1) establish the extent of opportunities for members of the public to check their own blood pressure (BP) outside of healthcare consultations (BP self-screening), (2) investigate the reasons for and against hosting such a service and (3) ascertain how BP self-screening data are used in primary care.

**Design:**

A mixed methods, cross-sectional study.

**Setting:**

Primary care and community locations in Oxfordshire, UK.

**Participants:**

325 sites were surveyed to identify where and in what form BP self-screening services were available. 23 semistructured interviews were then completed with current and potential hosts of BP self-screening services.

**Results:**

18/82 (22%) general practices offered BP self-screening and 68/110 (62%) pharmacies offered professional-led BP screening. There was no evidence of permanent BP self-screening activities in other community settings.

Healthcare professionals, managers, community workers and leaders were interviewed. Those in primary care generally felt that practice-based BP self-screening was a beneficial activity that increased the attainment of performance targets although there was variation in its perceived usefulness for patient care. The pharmacists interviewed provided BP checking as a service to the community but were unable to develop self-screening services without a clear business plan. Among potential hosts, barriers to providing a BP self-screening service included a perceived lack of healthcare commissioner and public demand, and a weak—if any—link to their core objectives as an organisation.

**Conclusions:**

BP self-screening currently occurs in a minority of general practices. Any future development of community BP self-screening programmes will require (1) public promotion and (2) careful consideration of how best to support—and reward—the community hosts who currently perceive little if any benefit.

**Strengths and limitations of this study**

- Using a mixed methods approach enabled us to gain an overview of BP self-screening opportunities within an area and an in-depth insight into the views of current and potential service hosts.
- GPs, practice managers and pharmacists were interviewed about existing BP self-screening and professional-led services.
- There was limited response from hosts of potential community-based BP self-screening schemes to the survey and interviews perhaps reflecting a lack of public interest or unease in talking about the topic.

## Background

National surveys have tracked the improvement in the levels of awareness, treatment and control of hypertension in England over the last two decades.^1^ However, these remain suboptimal, especially when compared with countries such as Canada.^2^ While the prevalence of untreated hypertension in England has dropped from 20% of men and 16% of women in 2003 to 14% of men and 11% of women in 2011,^3^ a significant number of people remain at increased risk of cardiovascular disease.^4^

The first step in receiving appropriate treatment is detection. Blood pressure (BP) screening has been traditionally carried out opportunistically in primary care consultations by healthcare professionals. Self-screening—whereby a patient not known to have hypertension checks their own BP outside of such a consultation—may provide a means to improve accessibility to BP screening and reduce undetected hypertension.^5^ Unlike self-monitoring, self-screening may involve a one-off measurement and therefore individuals may tend to use open access monitors placed in communal areas, rather than purchasing a monitor for regular measurement at home. Proposed benefits include reduced healthcare professional workload^6^ and the removal of the white coat effect but these are dependent on self-screening devices being clinically validated and producing accurate results.^7^

Non-physician screening—such as BP checks offered by pharmacists^8^—may also provide an alternative to opportunistic screening in primary care consultations. A recent systematic review of self-screening and non-physician screening found that community-based screening schemes can detect raised BP, which may in turn lead to the identification of new cases of hypertension.^5^ The review found examples of screening being conducted in pharmacies, public areas and retail spaces, community buildings such as fire stations, places of worship, mobile screening units, dental practices as well as self-screening conducted at the health centre. However, the authors concluded there was currently insufficient evidence to recommend specific approaches, and that studies with good follow-up of patients to definitive diagnosis were needed.

The Quality Outcome Framework (QOF) pay for performance system, introduced in 2004, for primary care in England and Wales recognises the importance of checking BP and incentivises general practices to undertake such activity.^9^ Solid cuff BP monitors—designed for unsupervised patient use—are being promoted by the manufacturers as a way of achieving QOF BP screening targets and are increasingly being found in the waiting rooms of general practices.^6^ Little is known about how these BP monitors are used by primary care staff or patients.

As part of a programme of research investigating the feasibility of community-based BP self-screening, we set out to establish the extent of BP self-screening and non-physician screening opportunities available in Oxfordshire, UK. We also aimed to examine the reasons for and against providing such a service, and how the existing services are used on a day-to-day basis. A linked paper reports the views and experiences of members of the public regarding such facilities. (Tompson *et al*, in press, BJGP).

## Methods

To answer the study objectives, a mixed methods study comprising of a survey and series of semistructured interviews was undertaken.

### Survey

Using a sampling frame informed by the community locations identified by the BP self-screening systematic review,^5^ and suggestions from local clinicians and healthcare commissioners, a telephone survey was conducted in summer 2013. Questions were designed to elucidate the extent and form of BP self-screening facilities, and included an open-ended question to allow identification of any additional sites. The survey was split into three phases for pragmatic reasons.

In the first phase, all Oxfordshire general practices, pharmacies and dental surgeries listed on the NHS Choices website were telephoned, apart from those offering specialist services (such as online pharmacies). In the second phase, religious organisations based in Oxford City only and listed in the Oxford Daily Info Directory (http://www.dailyinfo.co.uk) were emailed (due to their limited telephone cover). Council leisure centres and branches of national chain gyms across Oxfordshire were also surveyed as part of phase two. BP checks conducted as part of the gym induction process were not classified as open access BP screening as they were not available to the general public who did not want to join the gym. If there were any positive responses in phase two, phase three would be initiated with local—rather than national—gyms in Oxfordshire and religious organisations outside of Oxford City also being surveyed.

Survey data were analysed descriptively in SPSS V.21. The practice demographics (number of general practitioners (GPs), number of registered patients) and performance (total QOF score across all areas, hypertension register size, prevalence of hypertension, estimated prevalence of undiagnosed hypertension, proportion of patients with hypertension with a BP measurement in the last 9 months, proportion of patients with hypertension with controlled BP and patient rating surgery good or very good) were collated from routinely collected data and compared using the Mann-Whitney U Test as the QOF data were negatively skewed. The deprivation score for the postcode of each GP practice based on the 2010 Index of Multiple Deprivation was also compared.^10^

### Interviews

A series of semistructured interviews were conducted with existing and potential hosts of BP self-screening services to explore the reasons for and against providing such a service. All Oxfordshire pharmacies were invited to participate; due to the limited response rate, all those that responded were interviewed. General practices were categorised based on their BP self-screening status and a purposive sample recruited. Potential community sites, as defined in the systematic review,^5^ were also purposively sampled to ensure that a range of locations and types of community premises were studied and invited to participate.^11^ ^12^

Semistructured interviews were conducted face-to face or over the telephone by a non-clinical researcher trained in conducting in-depth interviews. They were digitally recorded, transcribed and checked for accuracy. An interview schedule was developed informed by the study objectives and refined following initial use (see online supplementary file one). Topics covered include the reasons for and against providing BP self-screening and how BP screening services are operationalised. Other relevant issues raised by interviewees were also explored.

10.1136/bmjopen-2016-013938.supp1 supplementary file

NVIVO (QSR International) software was used to organise the transcripts and coding process. A framework approach was taken to enable anticipated and novel themes emerging from the data to be identified.^13^ A coding framework was developed and refined by constant comparison.^13^ The One Sheet of Paper analysis method was used which involves listing on a single sheet all the issues which contribute to a single code and grouping these to form initial themes^14^ that were developed by discussion between researchers undertaking the analysis (AT, AW).

### Consent

All interviewees gave written informed consent and received a gift voucher from the research team as a token of their gratitude.

## Results

### Survey

At the time of the survey, there were 82 general practices delivering services on 99 sites in Oxfordshire. Eighteen practices (22%) offered BP self-screening (table 1), three of which shared a building and a BP self-screening monitor. One practice had monitors for BP self-screening in their main and branch surgeries. In addition to the 18 practices, another had recently completed a trial loan period of the monitor but had been unable to continue due to its cost, while another two practices were actively considering purchasing a self-screening monitor. Comparison of the characteristics of practices with or without the equipment did not reveal any statistically significant difference (see online supplementary file two).

**Table 1:** Locations offering BP self-screening

	Number approached	Number (%) that completed survey	Number that offered BP self-screening (%)	Number that offered professional-led BP screening (%)
General practices	82	82 (100.0)	18 (21.9)	*
Pharmacies	110	110 (100.0)	0 (0.0)	68 (61.8)
Dental practices	88	80 (90.9)	0 (0.0)	0 (0.0)
Leisure centres/gyms	26	26 (100.0)	0 (0.0)	0 (0.0)
Religious groups	42	27 (64.3)	0 (0.0)	0 (0.0)

*BP screening via professional medical staff was available at all general practices but required an appointment with a doctor or nurse.

BP, blood pressure.

10.1136/bmjopen-2016-013938.supp2 supplementary file

No pharmacies offered BP self-screening; however just over 60% offered measurements conducted by staff. Chains of pharmacies tended to either all offer BP checks or not. One supermarket pharmacy chain only offered BP checks during specific campaign weeks. Just over half of the independent pharmacies offered BP checks (20/37, 54.1%). Of the 80 dental practices that completed the survey, none provided facilities for their patients to check their own BP.

None of the religious groups emailed offered BP self-screening. The private gyms reported that members could request BP measurements to monitor the effects of training programmes. Council-owned gyms participated in the GP exercise referral scheme which required them to check participants' BP. Neither of these activities met the definition of BP self-screening. Phase three of the survey was not initiated.

### Interviews

In total, 23 interviews were conducted—table 2 describes the interviewee characteristics. Three were carried out over the telephone for logistical reasons. Emerging themes were organised into six headings.

**Table 2:** Characteristics of interviewees

	n	Interviewee details
Primary care (n=8)*		
Current BP self-screening providers	5	General practitioners (3) Practice managers (2)
No or discontinued BP self-screening	2	General practitioners (2)
BP self-screening in set-up	1	Practice manager (1)
Pharmacy (n=5)		
Professional-led BP screening only	4	Pharmacists (1 independent, 2 chain), pharmacy assistant (chain)
No BP screening available	1	Pharmacist (independent)
Community (n=10)		
Professional-led BP screening only	1	Housing association worker
No BP screening available	9	Shopping centre manager, supermarket worker, optician, community centre manager, day centre volunteer, head teacher, librarian, advice centre volunteer, charity volunteer
Other sites/organisations approached to participate but did not reply:		
Social clubs (2), leisure centres (3), rotary club (3), Know Your Numbers campaign (1), minority ethnic community groups (4).		

*all offered professional-led checks.

BP, blood pressure.

### Primary care responses

Interviewees represented eight general practices located across the county.

#### Helping with workload?

Primary care interviewees framed the BP self-screening equipment as assisting with the workload of checking BP—particularly those checks triggered by administrative reminders—rather than as a tool to reduce undetected hypertension: “You know about QOF pop-ups and things? …‘This person hasn't had their blood pressure checked for 5 years’ and they're somebody that never comes to the practice and you really want to catch them and do it then, but you're already running half an hour late…” (GP, Practice E with BP self-screening, (BPSS)). While it was acknowledged that checking BP was good practice, it contributed to the workload of already busy surgeries: “QOF want BP measurements for pretty much everybody … it adds, you know, another few minutes to every single patient … clinician time is always at a premium” (Practice Manager (PM), Practice Bl, BPSS in set-up). BP self-screening was felt to help free up time in consultations and the number of patients requiring appointments specifically to get their BP checked: “They [the GPs] are happy because, like I said, they can send the patient straight out and say, ‘Go check your blood pressure’ rather than trying to find an appointment with a nurse or a health care assistant” (PM, Practice W with BPSS).

The perceived benefits on clinician workload and also its ability to detect undiagnosed hypertension were largely unaudited: “I don't have any ideas about screening… it's just anecdotal, I don't know” (GP, Practice S with BPSS)*.* For some, there had been concerns prior to operationalisation that self-screening could actually increase the number of measurements taken in consultations: “We thought…we're going to get inundated with slightly iffy results and we're going to end up checking it more but that's not been the reality” (GP, Practice Wg, with BPSS). One GP, whose practice had decided against getting the equipment, was concerned that the stress of publically measuring BP in the waiting room would cause inaccurate readings. This, in turn, could cause unnecessary patient anxiety: “It had the potential to kind of sabotage reasons that people might be coming to their GP, if their primary concern had been something else but they came and then had a high blood pressure reading, for all sorts of silly reasons” (GP, Practice C, without BPSS).

#### Useful information?

Within our sample, there was variation in what the GPs were willing to use the self-screened results for. For screening purposes, all used it as a ‘rule-out device’ but would repeat elevated self-screened results themselves: “If they've then got a low result I'll be happy to accept that and put it into the computer… whereas if it's high I would always check it myself again before making any treatment decisions on the basis of that result” (GP, Practice E with BPSS).

Some surgeries used the waiting room monitors largely for the management of patients with hypertension. These readings were nested among office measurements—on the basis of which treatment was initiated—and, for some patients, alongside home monitoring. There was uncertainty regarding the comparative accuracy of self-screened BP measurements: “My perception… is that the results are usually lower when they do them in the waiting room and probably more accurate, so more closely tied to home readings” (GP, Practice E with BPSS); “I would put, put it closer to an office reading…. you're in in a medical environment” (GP, Practice C without BPSS). Owing to uncertainty about the accuracy of self-screened results, including patient adherence to the measurement protocols, one GP preferred to repeat all measurements taken in the waiting room when monitoring patients with hypertension thus negating any potential timesaving benefits: “It takes a minute to check someone's blood pressure. So, I mean, I'll be checking it anyway whatever that [the waiting room monitor] says” (GP, Practice Wg with BPSS).

There was also doubt about how many repeat measurements should be taken: “Some patients just have a single one and some patients do it three times and then you end up with three little slips of paper. And there aren't instructions for that….it's a bit kind of random” (GP, Practice S with BPSS). Some practices requested three readings while others used a single measurement: “One of the other practices … had a big list by the side of it that said, ‘If your blood pressure is at this point wait fifteen minutes and take another reading. If your blood pressure is at this point do nothing, come back again in a week's time and try again’ and all these things and we thought the more complicated you make it…, the more problems you get …So we looked at it very simple, take one” (PM, Practice K with BPSS). Practices had reached different conclusions regarding the trade-off between minimising measurement burden and maximising the accuracy of readings.

#### Empowering patients—an opportunity missed?

Providing facilities for patients to check their own BP was recognised as an opportunity for health promotion and patient education: “Obviously people should ideally know their numbers and by that I mean be informed about what's normal” (GP, Practice S with BPSS). However, this was a secondary benefit when compared with reducing workload. In spite of the opportunity to improve patient knowledge, there was limited provision of information alongside the monitor: “We haven't thought about doing that I suspect that would come under the heading of a little knowledge is a dangerous thing” (PM, Practice Bl BPSS in set-up). There was very limited offsite promotion meaning that only patients already attending—and engaged with—primary care were aware of the facilities to check their own BP.

#### Location: privacy versus accessibility

Taking BP measurement outside of its traditional setting of a 1:1, private healthcare consultation triggered reflection of where best to place the BPSS equipment: “*…* a personal service going on in a public waiting room… we just felt slightly uneasy with that” (GP, Practice C without BPSS.). There was no one ideal location within the conventional spaces of a GP surgery: “We really just put them where we could find space that's a bit private. So we've got one with a seat at the bottom of the stairs…a kind of a cubby hole” (GP, Practice S with BPSS). There were mixed views about the use of partitions to screen service users: “We're going to buy a screen cos currently it's just open in the waiting room so anyone can see you sitting there sticking your arm in the machine and we've listened to, sort of, patient suggestions” (PM, Practice W with BPSS); “When it was behind a screen … it was almost more off putting to go in” (GP, Practice Wg with BPSS).

#### Pharmacy checks—part of the job

Five interviewees represented four pharmacies, three of which offered professional-led BP checks. For pharmacies, provision of BP checks was linked to their dual identity as a place of healthcare and as a retail space; although a commercial enterprise, it is one with a social conscience: “You do it [BP check] as a kind of service to the community that pharmacies offer. But, just like we give free advice all the time and stuff like that, it's part of the job” (pharmacist Bb with BP checks). BP checks were felt to be exempt from the business model as they were of public health value. Although pharmacists liked providing the service—“I think it's actually quite a nice thing for the pharmacist to do. Breaks up the routine of the day, doesn't it a bit?” (pharmacist Bc with BP checks)—its promotion was limited due to lack of direct commercial benefits.

Pharmacy BP checks were triggered by physical symptoms or conducted as part of medication review appointments: “I tend not to push it on them. Most people come to me and say, ‘I'm not well and I think my blood pressure’ or whatever then I would do it” (independent pharmacist O with BP checks); “I'll tie them in with MURs [medicine usage reviews] …‘How often do you have your blood pressure checked?’ … Would you like to me check it now?’”(pharmacist Bc with BP checks). There was little evidence of asymptomatic members of the public requesting to have their BP measured, that is, for screening purposes.

Different service models were used—some pharmacists conducted the whole process: “If I let anyone else do it, I then can't see the patient and find out a bit more. I would have to waste time by asking someone to tell me what was said” (independent pharmacist O with BP checks). In others, the measurement was conducted by an assistant following a protocol: “If it's a really high one, I tend to get [the pharmacist] to do the advice which is his job rather than mine” (pharmacy assistant Bb with BP checks). Interpreting the reading was recognised as requiring skill: “Any idiot can do blood pressure readings, it's understanding what it is like” (pharmacist O with BP checks).

For one smaller pharmacy, a lack of staff, training and suitable space were barriers to providing a BP screening service: “I'm the only pharmacist here. I just have two members of staff who are trained to work in the medicines counter but nothing else” (pharmacist Bf without BP checks). The pharmacist also acknowledged the local general practice's library of home and ambulatory BP monitors available for patient use: “that's certainly way beyond anything I can offer” (independent pharmacist Bf, without BP checks). It was felt starting a pharmacy-based service may cause duplication of effort and the potential to upset the local practice: “Until they [the clinical commissioning group] express a definite desire for this to happen then I'm steering out of it.” (pharmacist Bf, without BP checks).

#### BP self-screening in community locations—an odd thing to see?

Apart from some pharmacies and the health bus, no other community sites offered BP checks. Interviews revealed that several were involved in other health-related activities, for example, a mental well-being self-help reading list at the library and healthy eating events at the supermarket. These, however, had a tangible link to the site's raison d'etre*,* something that was harder to demonstrate with BP screening*:* “It would be an odd thing to see a blood pressure machine apropos of nothing, you know, without some sort of context to it” (community centre manager without BP checks); “We're very limited for space. Space is for selling which is ultimately the main thing, reason, we're here” (supermarket representative without BP checks).

While community interviewees expressed an interest in promoting well-being and neighbourhood involvement, it was unclear what benefits hosting BP screening would provide them: “It might bring more people [in] but I'm sceptical about that” (advice centre representative without BP checks). The organiser of the health bus—the one community location that did offer BP measurements—described limited public interest and the effort required to drum up service users: “It's hard slog, you know, people don't just come rushing out and say ‘Oh yes, please take my blood pressure!’” (community worker with BP checks).

Interviewees reported minimal health service impetus to set-up BP screening services with no sites having been approached by healthcare commissioners. For sites with no history of providing BP checks, there was no experience or expertise to draw on: “They [service users] would start asking me questions that I wouldn't be confident to answer” (Daycentre worker without BP checks). These concerns were echoed by others: “Would it be an extra responsibility for us? Or would somebody come and service it or look after it… or do we need to train people” (optician without BP checks). There were also concerns that a poor self-screening experience may reflect badly on their organisation.

Table 3 summarises the findings of the studies and areas of uncertainty regarding BP self-screening identified.

**Table 3:** A summary of study findings

Screening type	Self-screening		Professional-led screening
Location	Within medical facilities	Non-medical facilities	
	GP surgery waiting rooms	Community locations	Pharmacy
Perceived benefits	Reduces GP workload	A service to the community	A service to the community
	Useful as a rule-out device	Raises awareness of BP screening	Adds variety to pharmacists' working day
	Improves screening attainment		
	Trained personnel available to advise, reassure patients		Trained personnel available to advise, reassure patients
Uncertainty regarding placing BP self-screening kiosks in these locations	Accuracy of measurements	Accuracy of measurements	Accuracy of measurements
	Measurement protocol	Measurement protocol	Measurement protocol
	Acceptability to members of the public (manuscript in preparation)	Health service commissioner/primary care demand	Health service commissioner/primary care demand
		Acceptability to members of the public	Acceptability to members of the public
		Benefits to the host venue	Financial benefits to venue
		Ability of members of the public to interpret their results	
		Ensuring appropriate follow-up	

BP, blood pressure; GP, general practice.

## Discussion

### Summary of main findings

We found no evidence of BP self-screening activities outside of GP surgeries. Within general practice, there was a general feeling that within surgery BP self-screening is a beneficial activity that reduces clinician workload and improves attainment of performance targets. However, variation within our sample revealed uncertainties regarding the utility of self-screened BP measurements in patient care. Although the pharmacists interviewed enjoyed checking BP, without direct financial reward they were unable to develop the service further. Among other potential hosts, barriers to providing a BP self-screening service included a lack of healthcare commissioner and public demand and a weak—if any—link to their core objectives as organisations.

### Comparison with existing literature

A systematic review found a paucity of data regarding the impact of BP self-screening on the detection of hypertension and the prognostic accuracy of self-screened readings.^5^ This was also reflected in the primary care interviewees' narratives. Before self-screening could be recommended more widely, robust studies are needed evaluating the impact of self-screening on clinician workload and hypertension detection. This would enable GP surgeries and other potential providers to make an informed decision about the value of such equipment and whether it should be deployed in community locations.

Another area of uncertainty raised by interviewees was the accuracy of self-screened measurements. One recent study found that BP measurements taken in the waiting room were comparable with ambulatory BP monitoring,^15^ which is regarded as the gold standard measurement. This was using the BpTRU device, however, rather than one specifically designed for waiting room use and each patient took multiple readings in contrast with the ad hoc practices we recorded. Studies of waiting room monitors in obstetric clinics found that women rarely followed the measurement instruction;^16^ ^17^ however, the impact on the accuracy of the resulting measurement was not assessed.

Our linked paper explores the patient experience of self-screening (Tompson *et al*, in press, BJGP). Users of GP waiting room monitors liked the experience and felt reassured by its location within a medical facility. Non-users expressed doubts about their ability to measure and interpret their BP as non-clinicians. There was a lukewarm response to the idea of community self-screening stations: some felt it would increase awareness regarding BP screening while others felt its unsupervised nature could cause anxiety.

We found no evidence of self-screening stations in community settings. We are aware of only one published example of self-screening in the UK.^18^ Hamilton *et al* placed 13 monitors in community locations and found that over a period of around 8000 hours in total during which time 759 first-time users were recorded, reflecting the limited public demand reported in our study. In Hamilton's study, 1.4% of service users were subsequently diagnosed with hypertension and the authors concluded that self-screening of BP was feasible in terms of “machine placement, functioning and durability, and user acceptability, and to have a reasonable impact on primary care”.

During the survey, we found evidence of non-permanent BP screening opportunities. The national ‘Know Your Numbers Campaign’ coordinated by the Blood Pressure Association holds screening events each September run by volunteers including healthcare professionals. In 2013, the results for 129 people who had their BP checked in Oxfordshire as part of this initiative were received by the campaign headquarters.^19^ A local housing association provided a weekly health bus that visits a housing estate. Residents could refer themselves to receive an on-board health check at an appointment with a nurse that includes a BP measurement. Both of these examples of ‘pop-up’ community-based BP screening relied on screeners to encourage people to be measured, to take the measurement and interpret the reading on behalf of service users.

### Strengths and weaknesses

Our mixed methods allowed an in-depth insight into the extent of current BP self-screening services in an area of the UK, their clinical utility and opinion regarding hosting a BP self-screening service.

BP self-screening is one feature of the ongoing process of devolving hypertension management away from traditional medical providers.^20^ Another is the introduction of nurse-led hypertension clinics.^21^ For pragmatic reasons, it was not possible to include practice nurses in our sampling frame but their experiences may have provided an alternative, valuable view point into the use of waiting room BP monitors.

Furthermore, not all the community hosts approached responded to our invitation to be interviewed and so while thematic saturation was reached among the 10 community interviews, further opinions may have been missed. The difficulty in recruitment may reflect a lack of public interest in the topic or perhaps a feeling of being underqualified to about what is viewed as a medical problem.

### Implications for practice

Our findings suggest that existing self-screening systems in primary care are geared towards checking the BP of patients who are in the surgery because they have an appointment. Increased promotion of BP self-screening facilities could help raise awareness among those that infrequently attend primary care, improving equity of access and also achieving pay-for-performance targets.

Among our sample, GP surgeries struggled to find an appropriate space for BP self-screening activities. When designing new health centres, consideration should be given to this recent addition to the type of work conducted in primary care, allocating areas which balance privacy with accessibility to maximise acceptability.

### Recommendations for further research

If BP self-screening is to be widely adopted, studies investigating its accuracy compared with office or home measurements are needed in order to maximise its utility to GPs and patients^22^ ^23^ as has been investigated for BP measurements taken by patients.^24^ Furthermore, there was uncertainty about how many repeat measures are required during a screening session to optimise accuracy versus unnecessary measurement burden on patients.

Given our findings, we are unable to currently make recommendations for the widespread implementation of BP self-screening. Table three summarises our results and highlights areas of uncertainty regarding BP self-screening. While taking BP screening outside of the relatively controlled environment of the GP, surgery may increase public awareness and accessibility; it makes maintaining the monitors and ensuring appropriate primary care follow-up more problematic.
