# Supplementary material for: Current and potential providers of blood pressure self-screening: a mixed methods study in Oxfordshire
Source: BMJ Open. 2017 Mar 22;7(3):e013938. doi: 10.1136/bmjopen-2016-013938 (PMC5372057; doi:10.1136/bmjopen-2016-013938)
Supplement: supplementary file [file bmjopen-2016-013938supp1.pdf]

Blood Pressure Self-Screening in Oxfordshire
Potential and Current Service Providers Interview Topic Guide

General

Can you describe your premises?
What is the neighbourhood like?
What is your role and responsibilities?
Do you currently provide facilities for people to check their blood pressure?

Providers that do currently offer screening

How did you come to offer blood pressure screening?
What was it like setting up the service?
What training did staff receive about the service?

How does the service work?
Prompts:

- *Who does the screening?*
- *Does it impact "normal" workloads?*
- *Where does the screening take place?*
- *Do you have sufficient equipment?*
- *How long does a blood pressure check take?*
- *How do most people hear about the service?*
- *Following the initial set up of the service, are any problems encountered on a day-to-day basis?*
- *Have you received any feedback about the service from staff or service users?*

What happens to follow-up raised blood pressure results?
Prompts:

- *Who is responsible?*
- *Do you follow any guidance for interpreting/acting on results?*
- *How do you feel advising service users?*
- *How does it work leaving people to follow up their own results?*
- *What are the typical questions service users ask about their results?*

Do you include a cardiovascular risk assessment as part of the blood pressure check?

Are you reimbursed for providing the service?

Have you ever had any doubts about the accuracy of the results measured at your premises?
Prompts:

- *Are the blood pressure monitors calibrated?*
- *Do you routinely replace the blood pressure monitors?*

Is the service well utilised?

Overall has your experience of providing blood pressure screening been positive or negative?

Are there any benefits to providing the blood pressure screening service?

<u>Sites that don't currently offer screening</u>

Have you ever considered providing blood pressure self-screening facilities?

Prompts if yes:

Where did the idea originate?
Why did you decide not to?
Could any of these barriers to providing the service be removed?
What level of reimbursement would change your mind?

If you were approached about hosting providing blood pressure self-screening facilities what would be your response be?

Could you imagine blood pressure self-screening working at your site?

Prompts:

Do you have a suitable space?
In some screening services, the member of the public measures their own blood pressure whereas in others a member of staff conducts the measurement for them. Who do you think would do the measurements at your site?
How comfortable would you feel measuring your clients/member of the public's blood pressure?
How appropriate do you think it is to measure blood pressure in a [location]
In some screening services, the members of the public interpret their own blood pressure whereas in others a member of staff provides them with advice. Which do you think would work best at your site?
Staff may require training about blood pressure self-screening. How easy would it to be organise a training session at your site?
Do you think the blood pressure measurement equipment would be safe at your location?

Would reimbursement for providing the service affect your outlook at all?

Do you think offering blood pressure self-screening service would provide any benefits to you and your premises?

Can you imagine clients/ members of the public using blood pressure self-screening services at your premises?

Prompt:

What sort of person do you think would use it?

<u>All</u>

Do you have views about people checking their own blood pressure using monitors stationed in the community?

Prompts:

Are there any potential disadvantages/ advantages to service hosts?
Are there any potential disadvantages/ advantages to service users?
Are there any disadvantages/ advantages compared to healthcare professional screening of blood pressure?

Do you know of any other location where blood pressure self-screening is offered in your neighbourhood?

Is there anything else we haven't covered?
