# Supplementary material for: Current and potential providers of blood pressure self-screening: a mixed methods study in Oxfordshire
Source: BMJ Open. 2017 Mar 22;7(3):e013938. doi: 10.1136/bmjopen-2016-013938 (PMC5372057; doi:10.1136/bmjopen-2016-013938)
Supplement: supplementary file [file bmjopen-2016-013938supp2.pdf]

Supplementary file two: Comparison of GP practices with and without BP self-screening facilities

Variable	All (n=79)*			Practices Offering Blood Pressure Self-Screening (n=14)			Practices Not Offering Blood Pressure Self-Screening (n= 65)			P value #
	Median	IQR	Range	Median	IQR	Range	Median	IQR	Range	
**Demographics (2010)**										
GP head count (excluding assistants and registrars)[1]	5.0	4.0-8.0	1.0-12.0	6.0	4.8-8.0	3.0-12.0	5.0	4.0-8.0	1.0-12.0	0.359
Weighted IMD 2010[2]	10.1	7.8-17.8	5.4-34.6	9.3	7.3-14.9	6.0-21.8	10.3	7.2-18.9	5.1-34.6	0.314
Registered list size 2010[3]	8151.0	5047.0-10791.0	584.0-19782.0	8212.0	5969.5-12211.5	4297.0-18871.0	7472.0	4604.0-10465.5	584.0-19792.0	0.472
**Performance data (2010-2011)**										
Total QOF Points, scored across all domains as a percentage of those available %[3]	99.2	97.8-99.7	81.1-100.0	99.4	98.5-99.8	96.1-100.0	99.2	97.8-99.7	81.0-100.0	0.223
Number of patients on hypertension register	938.0	541.0-1333.0	79.0-2749.0	1095.5	782.3-1558.8	433.0-2376.0	830.0	520.0-1239.0	79.0-2749.0	0.140
Patients with hypertension in whom there is a record of the blood pressure in the previous 9 months: Underlying achievement, net of exceptions, %[3]	91.5	89.9-93-6	79.3-99.5	91.1	87.7-93.5	83.5-96.1	91.5	90.0-93.7	79.3-99.5	0.555
Patients with hypertension in whom the last blood pressure (measured in the previous 9 months) is 150/90 or less Underlying achievement, net of exceptions, %[3]	80.6	76.8-85.4	57.1-91.7	80.4	77.6-84.2	68.6-86.2	80.6	73.3-80.2	57.1-91.7	0.608
Practice hypertension prevalence based on practice register, %[3]	12.2	9.0-14.0	3.7-17.7	13.9	10.2-15.4	7.8-16.6	12.1	8.8-13.8	3.7-17.7	0.090
Estimated Prevalence of Undetected Hypertension, %[4]	11.1	9.8-12.4	7.0-15.0	11.3	10.5-11.9	8.0-13.0	10.9	9.8-12.7	7.0-15.0	0.867
**Practice Survey Ratings (2011-12)**										
Survey respondents rating surgery experience as very good/good, %	93.0	88.0-96.0	74.0-100.0	93.0	88.5-96.0	79.0-97.0	93.0	87.0-96.3	74.0-100.0	0.767

* There were 92 practices in total with 3 practices excluded from the analyses: 1 was a GP surgery providing service for homeless patients only, two borrowed the BPSS equipment purchased by a third practice (they shared a building.)

# test used = independent samples Mann Whitney U Test

Data Sources

1. Health and Social Care Information Centre. Number of GPs per registered and weighted practice population, 2010. Accessed via https://indicators.hscic.gov.uk/webview/
2. Department for Communities and Local Government. The English Indices of deprivation 2010. Accessed via: https://www.gov.uk/government/uploads/system/uploads/attachment_data/file/6871/1871208.pdf
3. Health and Social Care Information Centre. Quality Outcomes Framework Data 2010/11. Accessed via: http://qof.hscic.gov.uk/index.asp
4. Eastern Region Public Health Observatory Modelled Estimated Prevalence of Hypertension, December 2011. Accessed via http://www.apho.org.uk/resource/item.aspx?RID=111119
5. Department of Health. Results of 2011-12 GP Patient Survey. Accessed via: https://www.england.nhs.uk/statistics/2012/06/14/gp-patient-survey-june-2012/
